# MPV17 Mutations Are Associated With a Quiescent Energetic Metabolic Profile

**DOI:** 10.3389/fncel.2021.641264

**Published:** 2021-03-17

**Authors:** Sandra Jacinto, Patrícia Guerreiro, Rita Machado de Oliveira, Teresa Cunha-Oliveira, Maria João Santos, Manuela Grazina, Ana Cristina Rego, Tiago F. Outeiro

**Affiliations:** ^1^Faculdade de Medicina, Universidade de Lisboa, Lisboa, Portugal; ^2^Serviço de Neurologia Pediátrica, Hospital Dona Estefânia, Centro Hospitalar Universitário Lisboa Central-EPE, Lisboa, Portugal; ^3^Department of Experimental Neurodegeneration, Center for Biostructural Imaging of Neurodegeneration, University Medical Center Göttingen, Göttingen, Germany; ^4^Switch Laboratory, Center for Brain and Disease Research, Vlaams Instituut voor Biotechnologie (VIB), Leuven, Belgium; ^5^Switch Laboratory, Department of Cellular and Molecular Medicine, Katholiek Universiteit (KU), Leuven, Belgium; ^6^CEDOC – Chronic Diseases Research Center, Faculdade de Ciências Médicas, Universidade Nova de Lisboa, Lisboa, Portugal; ^7^Center for Neuroscience and Cell Biology, University of Coimbra, Coimbra, Portugal; ^8^Faculty of Medicine, University of Coimbra, Coimbra, Portugal; ^9^CIBB - Center for Innovative Biomedicine and Biotechnology, University of Coimbra, Portugal; ^10^Translational and Clinical Research Institute, Faculty of Medical Sciences, Newcastle University, Newcastle upon Tyne, United Kingdom; ^11^Max Planck Institute for Experimental Medicine, Göttingen, Germany

**Keywords:** Mpv17 mutations, mitochondrial depletion syndrome, mitochondrial dysfunction, protein mislocation, neurode generation

## Abstract

Mutations in the MPV17 gene are associated with hepatocerebral form of mitochondrial depletion syndrome. The mechanisms through which MPV17 mutations cause respiratory chain dysfunction and mtDNA depletion is still unclear. The MPV17 gene encodes an inner membrane mitochondrial protein that was recently described to function as a non-selective channel. Although its exact function is unknown, it is thought to be important in the maintenance of mitochondrial membrane potential (ΔΨm). To obtain more information about the role of MPV17 in human disease, we investigated the effect of MPV17 knockdown and of selected known MPV17 mutations associated with MPV17 disease *in vitro*. We used different approaches in order to evaluate the cellular consequences of MPV17 deficiency. We found that lower levels of MPV17 were associated with impaired mitochondrial respiration and with a quiescent energetic metabolic profile. All the mutations studied destabilized the protein, resulting in reduced protein levels. We also demonstrated that different mutations caused different cellular abnormalities, including increased ROS production, decreased oxygen consumption, loss of ΔΨm, and mislocalization of MPV17 protein. Our study provides novel insight into the molecular effects of MPV17 mutations and opens novel possibilities for testing therapeutic strategies for a devastating group of disorders.

## Introduction

Mitochondrial DNA depletion syndromes (MDDS) are autosomal recessive disorders characterized by a severe decrease in mitochondrial DNA (mtDNA) copy number in affected tissues. MDDS are one of the most common forms of childhood respiratory chain deficiencies (Sarzi et al., 2007a; Yamazaki et al., 2014). The clinical presentation can be very heterogeneous, with overlapping phenotypes, but MDDS are usually classified as purely myopathic, hepatocerebral, encephalomyopathic, and neurogastrointestinal (Suomalainen and Isohanni, 2010; Spinazzola, 2011).

Several genes are associated with MDDS, affecting mtDNA maintenance by three possible mechanisms: (i) impaired mitochondrial nucleotide (dNTP) synthesis [mutations in *TK2* (Saada et al., 2001), *SUCLA2* (Elpeleg et al., 2005), *SUCLG1* (Ostergaard et al., 2007), *RRMB2* (Bourdon et al., 2007), *DGUOK* (Mandel et al., 2001), *TYMP* (Nishino et al., 1999), ABAT (Besse et al., 2015), SLC25A4 (Echaniz-Laguna et al., 2012), and AGK (Mayr et al., 2012)]; (ii) deficient mtDNA replication [mutations in *POLG* (Naviaux et al., 1999), POLG2 (Young et al., 2015), *TWINKLE* (Sarzi et al., 2007b), TFAM (Stiles et al., 2016), RNASEH1 (Reyes et al., 2015), MGME1 (Kornblum et al., 2013), and DNA2 (Ronchi et al., 2013)], which leads to defects in mtDNA synthesis and repair; or (iii) defective mitochondrial dynamics [OPA1 (Amati-Bonneau et al., 2008), MFN2 (Rouzier et al., 2012), and FBXL4 (Antoun et al., 2015)]. All these mechanisms cause deficient turnover and/or segregation of mtDNA to daughter cells, leading to a decrease in its copy number. Ultimately, mtDNA depletion results in inadequate synthesis of respiratory chain complexes with consequent deficient energy production and organ dysfunction (Sarzi et al., 2007a; Spinazzola et al., 2009).

*MPV17* is one of the genes associated with the hepatocerebral form of MDDS (MTDPS6; NNH; MIM #256810). To date, ~100 affected individuals have been reported with *MPV17*-related disease, with a total number of 48 *MPV17* pathogenic variants described (Karadimas et al., 2006; Spinazzola et al., 2006, 2008; Nogueira et al., 2007; Wong et al., 2007; Navarro-Sastre et al., 2008; Kaji et al., 2009; Parini et al., 2009; El-Hattab et al., 2010, 2018; AlSaman et al., 2012; Merkle et al., 2012; Mendelsohn et al., 2013; Uusimaa et al., 2013; Bijarnia-Mahay et al., 2014; Choi et al., 2015; Dalla Rosa et al., 2016; Kim et al., 2016).

Despite some clinical heterogeneity, the most common clinical manifestations in patients harboring mutations in *MPV17* are infantile-onset hepatic and neurologic manifestations, with cerebral leukoencephalopathy in brain MRI and metabolic abnormalities, including lactic acidosis. Progressive liver failure is frequently the cause of death in early childhood. More rarely (around 4%), *MPV17* mutations have also been associated with late-onset neuromyopathy with multiple mtDNA deletions in skeletal muscle with minimal or no hepatic disease (Blakely et al., 2012).

The human *MPV17* gene encodes a 176 amino acid protein of 18 kDa. MPV17 was previously thought to be a peroxisomal protein (Zwacka et al., 1994) but was later found to localize in the inner mitochondrial membrane (El-Hattab et al., 2018). Although the function of MPV17 and the mechanisms leading to mtDNA dysfunction are still unclear, MPV17 was described as a non-selective channel that modulates mitochondrial membrane potential (ΔΨ_m_) (Antonenkov et al., 2015). Several factors regulate the gating properties of this putative channel, which is maintained closed in normally functioning mitochondria (Antonenkov et al., 2015). In damaged mitochondria, the MPV17 channel is prone to be fully open. Some point mutations in MPV17 are also predicted to produce a leaky channel, unable to maintain a high (normal) ΔΨ_m_. Furthermore, loss of MPV17 function causes a total loss of channel activity, increasing ΔΨ_m_ (Antonenkov et al., 2015). In both cases there is excessive ROS production (Binder et al., 2011; Zorov et al., 2014). Loss of MPV17 function results in failure of oxidative phosphorylation (OXPHOS) and in mtDNA depletion in affected individuals (Karadimas et al., 2006; Wong et al., 2007; Uusimaa et al., 2013). In MPV17^−/−^ mice (Viscomi et al., 2009) a reduced rate of mtDNA replication has been observed, due to dNTP insufficiency (Dalla Rosa et al., 2016).

To better understand the role of MPV17 in human disease, we investigated the effect of MPV17 *knockdown* in human cell lines and characterized the effect of selected *MPV17* mutations associated with MDDS on cellular and organelle localization, and on mitochondrial bioenergetics.

## Materials and Methods

### Plasmids

Human *MPV17* cDNA (a kind gift from Prof. M. Zevianni, Milano, Italy) was cloned with an HA C-terminal tag, in pcDNA3.1 (Invitrogen) as previously described (El-Hattab et al., 2018). The *MPV17* mutations were generated by site-directed mutagenesis using the QuickChange® Kit (Stratagene). The sequences of the primers used for mutagenesis are shown in Supplementary Table 1. Mutagenesis was confirmed by Sanger sequencing (Supplementary Material 2). MPV17-specific shRNA-pLKO plasmid (clone ID TRCN0000129921; Santa Cruz Biotechnology) was used to knockdown the expression of MPV17 in cell lines.

### Cell Culture and Transfections

Cell lines were cultured in DMEM (Dulbecco's modified Eagles's medium) (Invitrogen) supplemented with 10% (v/v) FBS (fetal bovine serum), 10 U/mL penicillin and 100 μg/mL streptomycin at 37°C, in a 5% CO_2_ humidified incubator. HEK293T knockdown cell line was supplemented with 2.5 mM pyruvate and 0.2 mM uridine. Cells were transiently transfected with the different constructs using FuGENE®6 transfection reagent (Roche), according to the manufacturer's instructions.

### Subcellular Fractionation

Standard methods were used to prepare total cell lysates, mitochondrial, and post-mitochondrial fractions from cultured cells. In brief, cells were harvested, washed, and resuspended in MIB (Mitochondrial Isolation Buffer [225 mM mannitol, 75 mM sucrose, 10 mM MOPS, 1 mM EGTA, 0,5% BSA, pH 7.2]. Cells were homogenized with a glass Pyrex homogenizer (type B pestle, 30 strokes). Unbroken cells, large plasma membrane and nuclei were removed by two centrifugations at 1,000 × *g* for 10 min. The resultant supernatant was again centrifuged at 8,000 × *g* for 15 min at 4°C to separate the mitochondrial fraction (pellet) from the cytosolic fraction (supernatant). Mitoplasts were isolated from mitochondria by digitonin treatment (Gallet et al., 1999).

### Western Blot Analysis

Protein extracts were separated in 15% SDS-polyacrylamide gel and transferred to nitrocellulose membranes. Equal amounts of protein were loaded - 80 μg for whole cell homogenate and for endogenous MPV17, 50 μg for mitochondria, 50 μg mitoplasts. Protein concentration was determined using BCA Kit (Pierce). Primary antibodies used were: goat polyclonal anti-MPV17 antibody (1:250, SantaCruz Biotechnology), anti-HA (1:1000, SantaCruz Biotechnology), and anti-Complex IV (1:5000, Invitrogen). The secondary antibodies were developed using ECL kit (Bio-Rad) and band intensities were quantified using ImageJ software.

### Viral Production

The shRNA was co-transfected with the packaging plasmid pCMV-dR8.9 (Addgene) and envelope plasmid pCMV-VSV-G (Addgene) using FuGENE®6 transfection reagent (Roche). Non-specific shRNA-pLKO plasmid was used as control. Infection of 293T cells using lentivirus was performed by exposing cells to the virus-containing medium and incubating for 4 h. Cells were maintained under selection conditions with puromycin. Stable knockdown cell lines were periodically checked by standard Western blotting, using MPV17 antibody. Rescue studies in MPV17 knockdown cells were carried out by overexpression of HA-tagged constructs of wild-type MPV17 and mutant MPV17 cDNA obtained by mutagenesis.

### Real-Time Quantitative PCR Analysis of Mitochondrial DNA Content

The relative mtDNA copy number was evaluated using quantitative real-time PCR (qRT-PCR). In brief, DNA samples were diluted to 0.4 ng/μL using Tris-EDTA buffer solution. For each sample, two primer pairs were used to amplify the tRNA Leu(UUR) gene - mtDNA and ß-2-microglobulin (ß2M) gene - nuclear DNA, in separate wells. The primers sequences and qRT-PCR amplification conditions were previously described (Venegas et al., 2011). The amplification for each sample was performed in a final volume of 10 μL, using the 2 × SYBR SuperMix (iTaq SYBR Green Supermix with ROX, BioRad) and the primer concentration used was 5 μM. All samples were run twice, in triplicate for both mitochondrial and nuclear genes using 7500 Fast Real-time PCR system (qRTPCR; PE7500 real-time PCR instrument; Applied Biosystems, Foster City, CA, USA). A negative and a positive control were also included in each run in order to verify possible contaminations and to act as intern calibrator of values, respectively. Standard deviations for the cycle of threshold value were accepted at 0.50. The results were analyzed with the 7500® v2.0.4 software (Applied Biosystems).

### Confocal Microscopy

Cells were grown to around 60% confluency on glass coverslips coated with poly-D-lysine (Sigma). Cells were then sequentially incubated with medium containing 200 nM MitoTracker Red CMXRos (Molecular Probes), fixed in 4% paraformaldehyde (PFA), permeabilized with 0.1% Triton-100, blocked in 10% normal goat serum and incubated with primary anti-HA antibody (1:100, SantaCruz Biotechnology). Next, cells were incubated with conjugated secondary antibody anti-rabbit Alexa Fluor 488 (1:1000, Invitrogen) and DAPI. Images were captured in a Zeiss LSM 510 META confocal microscope (Carl Zeiss) with 63x/1.4 oil immersion objective. Acquisitions were made using the MetaMorph software (Universal Imaging). The images were analyzed in ImageJ.

### Cellular Metabolic Activity Studies

Cellular metabolic activity was used as a measure of viability and was determined using resazurin (Sigma-Aldrich, St. Louis, Missouri, USA) assay in 96-well plates. Fluorescence intensity was measured (excitation 560 nm, emission 590 nm) in a microplate reader Tecan Infinite® 200 (Tecan, MÄnnedorf, Switzerland). The release of lactate dehydrogenase (LDH) into the culture media was used as a measure of cytotoxicity, and was performed according to the manufacturers' instructions (Sigma-Aldrich).

### Mitochondrial Respiration Studies

Wildtype (control) and MPV17-knockdown HEK293T cells were grown in standard conditions. KD cell line was supplemented with 2.5 mM pyruvate and 0.2 mM uridine. The assay was performed in the Seahorse Bioscience XF24. Oxygen Consumption Rate (OCR) and Extra-Cellular Acidification Rate (ECAR) measurements were measured under basal conditions and after the sequential injection of 1 μg/ml oligomycin, 0.5 μM of FCCP and 1 μM rotenone/antimycin A, allowing for the estimation of basal respiration, proton leak, maximal respiration rate, spare respiratory capacity and ATP production (Tan et al., 2004). ATP production is calculated from the decrease in respiration rate when ATPase activity is blocked, meaning the difference between de OCR before and after oligomycin injection. Proton leak is the respiration that remains after ATPase activity inhibition by oligomycin minus the non-mitochondrial respiration. Maximal respiration rate (MRR) is the difference between maximal mitochondrial respiration measured as OCR in mitochondrial uncoupled by FCCP and the non-mitochondrial OCR measured in mitochondrial exposed to rotenone/antimycin A. Spare respiratory capacity (SRC), an indicator of the bioenergetics capacity, is the difference between OCR-F and OCR-B. The respiratory control ratio (RCR) is an index of mitochondrial coupling and is calculated as the ratio between the OCR-F and OCR-O.

### ROS Measurement

The total ROS content of cells was assessed using 2',7'-dichlorodihydrofluorescein diacetate (H2DCF-DA). Fluorescence intensity was measured (excitation 495 nm, emission 527 nm). Mitochondrial superoxide production was monitored using the superoxide-sensitive probe MitoSOX™ Red (Molecular Probes). Fluorescence intensity was measured (excitation 510 nm, emission 580 nm). A microplate reader Tecan Infinite® 200 (Tecan, MÄnnedorf, Switzerland) was used.

### Measurement of Mitochondrial Membrane Potential (ΔΨ_m_)

Mitochondrial membrane potential was measured using JC-1 probe (Invitrogen), according to manufacturer's protocol. Fluorescence intensity was measured (excitation 560 nm, emission 595 nm for J-aggregates in healthy cells and excitation 485 nm, emission 535 nm for monomers in apoptotic cells) in the microplate reader Tecan Infinite® 200 (Tecan, MÄnnedorf, Switzerland). The ratio of fluorescent intensity of J-aggregates to fluorescence intensity of monomers was used as an indicator of cell health.

### Lactate and Pyruvate Measurement

L-lactate and pyruvate were measured in the culture medium with L-Lactate Assay Kit (Abcam, #ab65330) and Pyruvate Assay Kit (Abcam, ab65342), respectively, in 96-well plates, according to the manufacturer's instructions.

### Statistics

Statistical analyses were performed with GraphPad Prism (Version 7.0). One-way ANOVA test with Bonferroni *post-hoc* test were performed as shown. Values are plotted as the mean ± *SD*. *p*-Values are based on the following pattern: ns (not significant; *p* > 0.05), ^*^ (significant; *p* < 0.05), ^**^ (significant; *p* < 0.01), and ^***^ (significant; *p* < 0.001).

## Results

### Reduction of MPV17 Protein Levels Affect Cell Metabolic Activity

**Figure 1:**
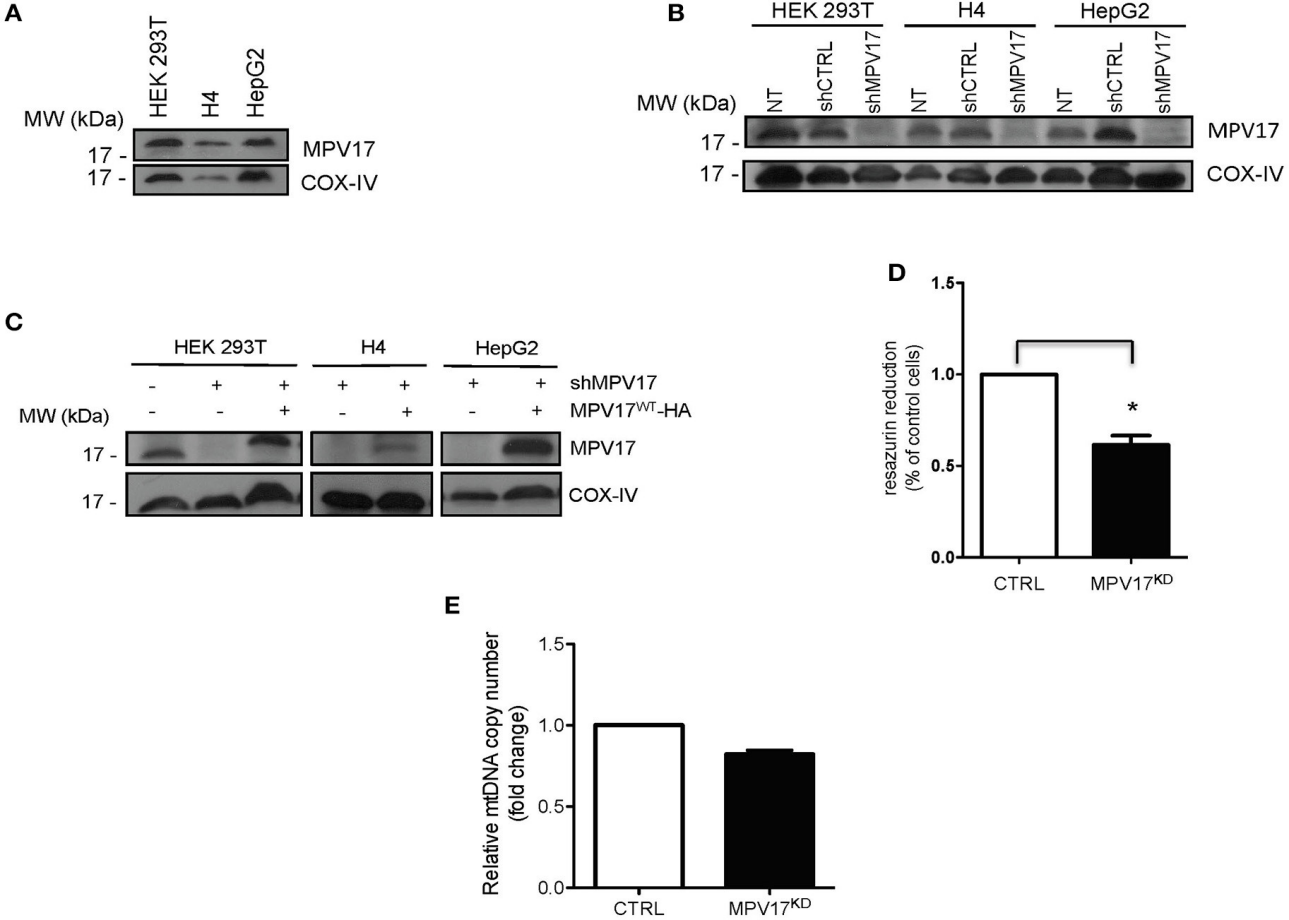
Expression of MPV17 in different cell lines. **(A)** Immunoblotting showing MPV17 endogenous expression in different human cell lines (HEK293T, H4 and HepG2) and **(B)** knockdown of MPV17 using shRNA system (shMPV17), when compared with shRNAcontrol (CTRL). NT - cells without any treatment. **(C)** Western blot showing that expression of MPV17-HA protein restores the protein levels in the different cell lines used. An anti-MPV17 antibody was used to detect endogenous MPV17 protein in mitochondrial fraction in three human cell lines. Anti-COX-IV antibody was used as a mitochondrial marker. Cells are plated at the day before experiments so that cells will be ~60% confluent on the day of transfection. Cells are analyzed 24 h after transfection **(D)** Effect of MPV17 knockdown on resazurin reduction capacity as a measure of metabolic activity. Wild-type HEK293T cells were used as control (CTRL) Cells were platted as 2 × 10^4^ cells/well and cultured for 48 h. **(E)** Effect of MPV17 knockdown on mtDNA copy number, determined by RT-qPCR. *n* = 3. *p < 0.05. Bars indicate the standard deviation (SD).

To investigate the molecular effects of *MPV17* loss of function, we developed a cellular model to analyse protein content, subcellular localization and mitochondrial bioenergetics. We assessed the endogenous levels of *MPV17* in three different cell lines by immunoblotting analyses. The protein was expressed at similar levels in HEK293T, H4, and HepG2 (Figure 1A). To generate an MPV17 knockdown (MPV17^KD^) cell line, we used a lentiviral vector encoded shRNA. In parallel, we generated a control cell line using a scrambled shRNA sequence. In the KD cells, the levels of MPV17 were efficiently reduced when compared to the control cells (Figure 1B). We restored MPV17 levels in the MPV17^KD^ cell line by transfecting wild-type MPV17 (Figure 1C; Supplementary Figure 3). Next, we assessed the metabolic activity of cells knocked down for MPV17 using the resazurin reduction assay. Under basal conditions, MPV17^KD^ cells showed decreased resazurin reduction by 38.5% when compared to control. As resazurin reduction can be used as a measure of cell viability but also as an indicator of overall mitochondrial function, this indicates MPV17^KD^ reduced cell viability and/or deficient mitochondrial oxidative capacity, as differences in cell proliferation were not detected by cell count (Figure 1D). Mitochondrial DNA (mtDNA) determination, using RT-qPCR, in MPV17^KD^ cells showed no significant difference when compared to cells expressing wild-type levels of MPV17 (Figure 1E).

### Reduction of MPV17 Protein Levels Decreases Mitochondrial Bioenergetics

**Figure 2:**
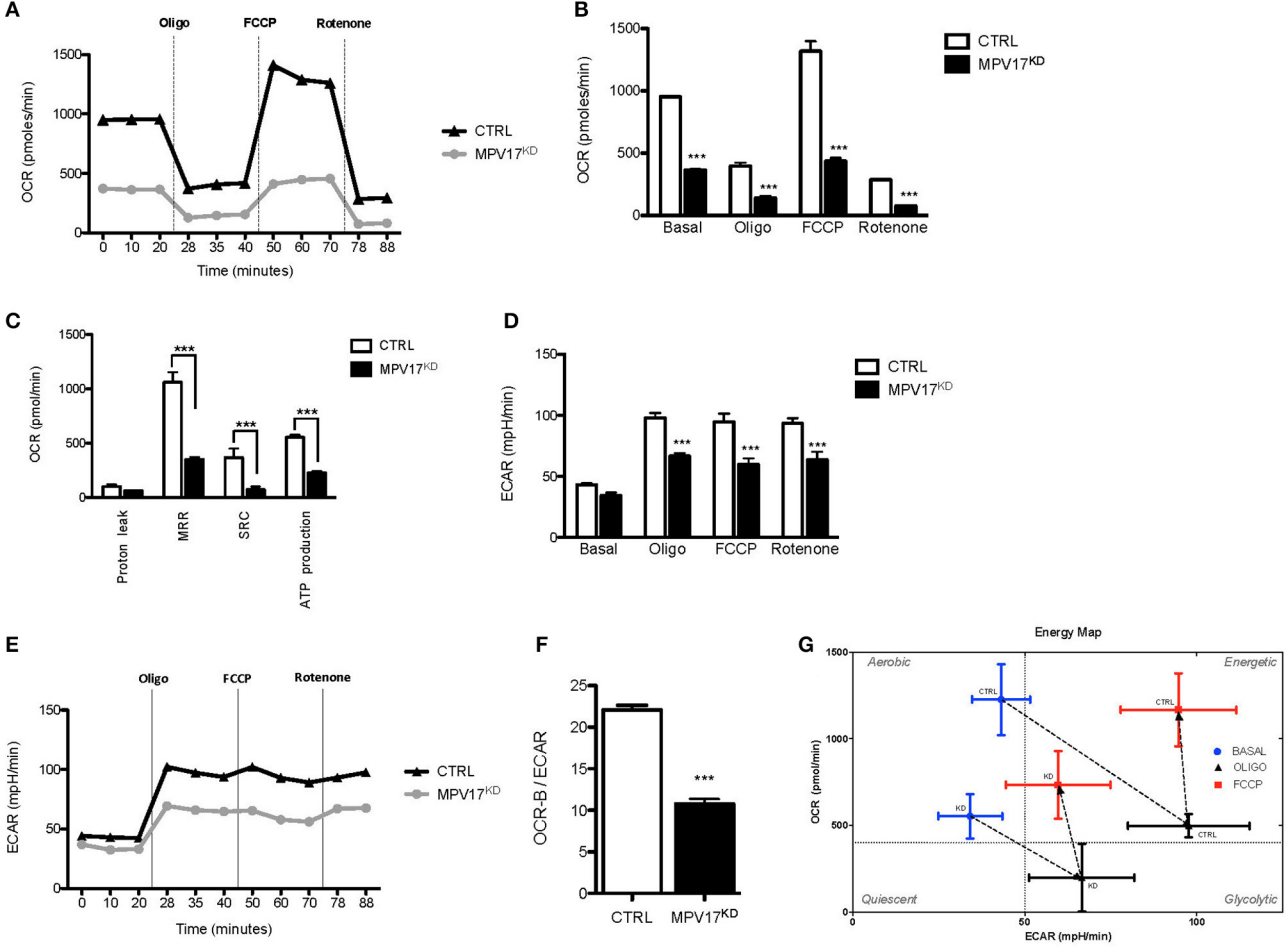
MPV17KD cells display reduced bioenergetics capacity. **(A)** OCR profile in HEK293T (CTRL) and knockdown (MPV17^KD^) cell lines. OCR expressed as pMolesO2/min. Vertical lines indicate the time of addition of oligomycin (Oligo), FCCP and rotenone/antimycin A, reflecting OCR-B, -O, and –F, respectively. MPV17KD cells are particularly insensitive to FCCP, indicating lower reserve respiratory capacity. **(B)** OCR in basal conditions, OCR-O, OCR-F, and OCR-R. **(C)** Individual bioenergetics parameters with maximal respiration rate (MRR), spare respiratory capacity (SRC) and ATP production. **(D,E)** ECAR profile in CTRL and MPV17^KD^ cells. The glycolytic activity after respiratory chain disruption with oligomycin and FCCP addition is lower in MPV17^KD^ cells than in control, expressed as lower ECAR. **(F)** Lower OCR-B/ECAR in MPV17^KD^ cells, reflecting insufficient increase in the rate of glucose utilization *via* anaerobic glycolysis to compensate mitochondrial respiratory deficiency. **(G)** Bioenergetic map of OCR-B/ECAR. The quadrants were set at arbitrary values to indicate only the direction of bioenergetics changes. All determinations were performed in 11 replicates for each cell line and the values were calculated after normalization to the final cell number and are plotted as the mean ± *SD*. ANOVA test for controls vs. MPV17^KD^. ***p < 0.001.

To understand how *MPV17* knockdown affects mitochondrial function, we analyzed the cellular respiratory profile using a Seahorse Flux Analyser, through the successive addition of oligomycin, FCCP and rotenone/antimycin A to block mitochondrial electron transport chain. In control conditions, oxygen consumption rate (OCR-B) is inhibited when ATP synthesis is blocked by oligomycin (OCR-O) and is stimulated when oxidation is uncoupled from phosphorylation by FCCP (OCR-F). OCR-B, -O, and –F were significantly lower (*p* < 0.001) in the MPV17^KD^ cells when compared with the control (Figures 2A,B). Values of OCR-B, -O, and -F were used to calculate other bioenergetics parameters, i.e., proton leak, maximal respiration rate (MRR), spare respiratory capacity (SRC), and estimate ATP production (Figure 2C). MRR, SRC, and ATP production were all significantly lower in the mutant cells when compared with control. Overall, these results indicate decreased mitochondrial bioenergetic capacity in MPV17^KD^ cells. Despite the slightly lower ECAR in basal conditions (Figures 2D,E), in stressful conditions (by the addition of oligomycin and FCCP) MPV17^KD^ cells showed lower ECAR when compared with WT (CTRL) cells. OCR-B/ECAR ratio was decreased in MPV17^KD^ cells (Figure 2F), and the bioenergetic map that plots OCR against ECAR reveal a limited glycolytic capacity of KD cells when compared with control. Overall, these results suggest that loss of MPV17 causes significant impairment in mitochondrial respiratory capacity and limited metabolic capacity to meet high energy demand (Figure 2G).

### MPV17 Mutants Maintain Mitochondrial Localization but Are Less Stable Than WT Protein

To investigate the effect of selected disease-associated mutations, we expressed MPV17 mutants in MPV17^KD^ cells. Considering the clinical criteria, and *in silico* analysis of the predicted effects of the amino acid substitutions, we selected five MDDS-related mutations for in depth molecular studies: arginine to glutamine substitution at position 50 (MPV17^R50Q^) which is the most frequent mutation; arginine to tryptophan substitution at position 50 (MPV17^R50W^) which usually associates with Navajo neurohepatopathy with a severe clinical prognosis; deletion of three amino acids encoding for Gly79_Thr81 (MPV17^79−81del^) which are predicted to delete a putative kinase C phosphorylation site; substitution of glycine for an arginine at position 94 (MPV17^G94R^) in the first amino acid of exon 5 which has a possible effect on splicing; and a substitution of a serine for phenylalanine at position 170 (MPV17^S170F^), localized at the C-terminal of the protein and possibly abolishes a phosphorylation site (Supplementary Material 2) Wild-type HA-tagged MPV17 (MPV17^WT^) was used as control.

**Figure 3:**
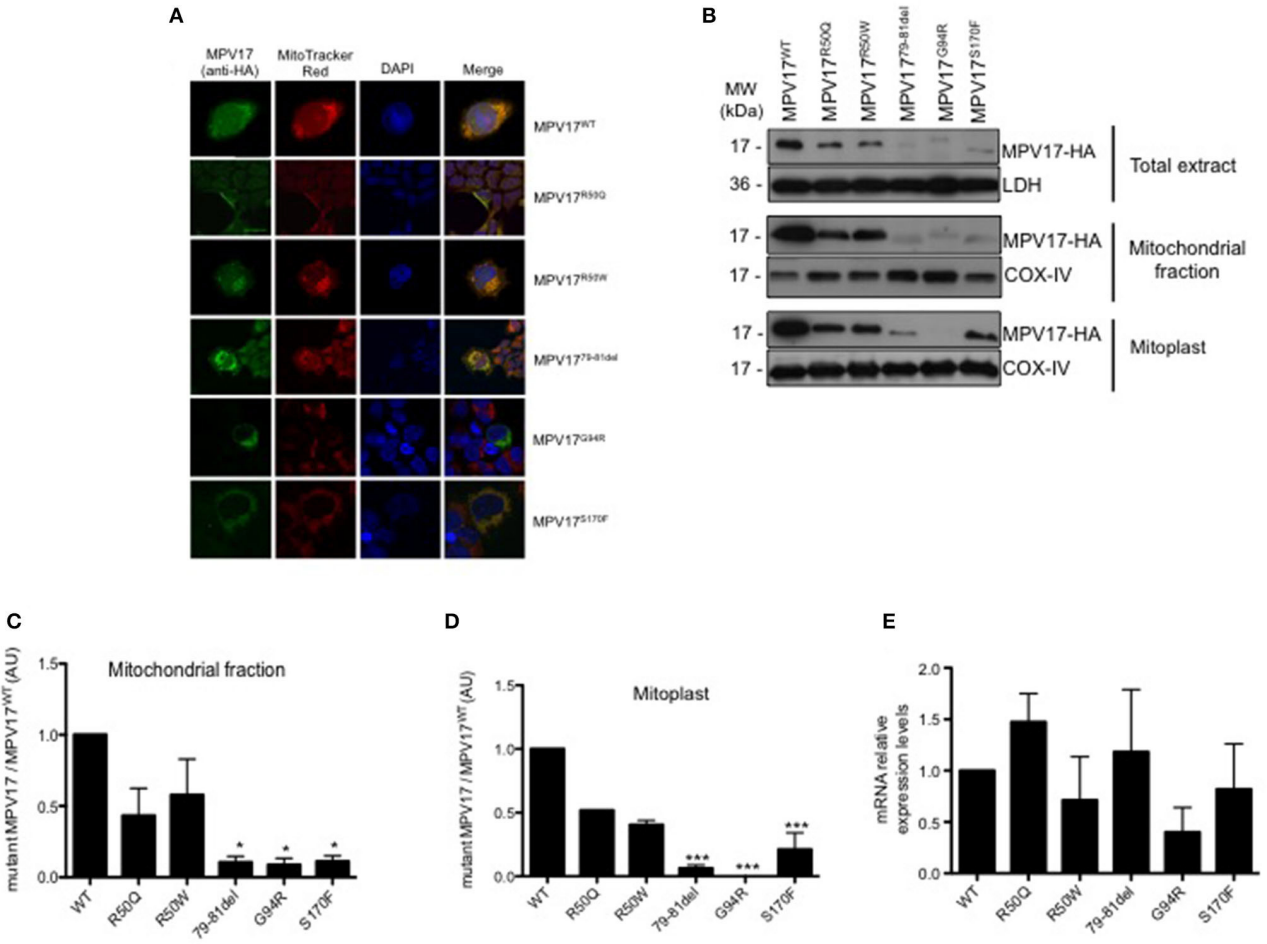
Expression and subcellular localization of MPV17 mutants. **(A)** Confocal microscopy images demonstrating that MPV17^WT^ (green) is distributed throughout the cytoplasm, and colocalizes with MitoTracker Red (red) in HEK293T transfected cells, indicating mitochondrial localization of the protein. In MPV17^G94R^ expressing cells, mitochondria do not stain with specific dye Mitotracker Red. Like wild-type protein, all other mutated versions of the protein, are localized in the mitochondria. Scale bar = 20 μm. **(B)** Representative immunoblotting of expression of MPV17-HA protein in HEK293T cells. **(C)** Quantitative histogram of both wild-type and mutant MPV17 protein enriched in mitochondrial fraction. All MPV17 mutant proteins have lower expression levels when compared with wild-type. **(D)** MPV17^G94R^ was not found in mitoplast, indicating abnormal localization inside the mitochondria. The intensity of the bands of Western blot, for each condition, and all the mutants were normalized by the intensity of the MPV17^WT^ (our control). LDH and COX-IV were used as cytosolic and mitochondrial markers, respectively. **(E)** The mRNA levels of MPV17 wild type and mutant genes were quantified by RT-qPCR. Values are reported as mRNA levels normalized respect to WT. Expression was normalized to the mRNA levels of the internal control actin (ACT1) (*n* = 3). ANOVA test for mutants vs. MPV17^WT^, bars indicate the standard deviation (*SD*), *p < 0.05 and ***p < 0.001.

To determine whether MPV17 mutations affect the subcellular localization of the protein, HEK293T knockdown cells were transfected with the various constructs and imaged using confocal microscopy. As expected, HA-tagged MPV17^WT^ displayed a punctate distribution in the cytoplasm that colocalized with the mitochondrial marker. Additionally, we observed mitochondrial localization for all mutant proteins, except for the MPV17^G94R^ mutant. In cells expressing the MPV17^G94R^ mutant protein, the mitochondria network was ill-defined or not staining with MitoTracker Red CMXRos, a ΔΨ_m_-sensitive dye (Tan et al., 2004). These mitochondria devoid of Mitotracker staining coexisted side by side with non-transfected cells with normal mitochondria staining. These results suggested that the MPV17^G94R^ mutation induced the dissipation of ΔΨ_m_ in the transfected cells (Figure 3A).

Although the mutations did not affect mitochondrial targeting of the protein, we hypothesized that they might affect the localization of the protein inside the mitochondria. To test this, we performed subcellular localization studies in cells expressing the different MPV17 variants. Both WT and mutant protein were enriched in the mitochondrial fraction (Figure 3B). However, MPV17 levels were markedly decreased for all mutants, especially for MPV17^79−81del^, MPV17^G94R^, and MPV17^S170F^ (<10% of levels found in MPV17^WT^) (Figure 3C). Mitoplasts were obtained by removing the mitochondrial outer membrane from isolated mitochondria. As expected, wild-type protein (MPV17^WT^) was found in mitoplasts, consistent with its inner mitochondrial membrane localization. In the presence of MPV17^G94R^ mutation, the mutant protein was not found in the mitoplast fraction (Figure 3D). All other mutations had no effect on the localization of the protein. To investigate if the reduced levels of mutant MPV17 protein were a consequence of decreased transcription, mRNA levels were assessed by RT-qPCR. When compared with WT, mutants mRNA levels are lower particularly for G94R but also for R50W and S170F mutants (Figure 3E), suggesting a compromised transcription for these mutants. The G94R mutant is mislocalized as well as transcribed at low levels when compared with the WT protein.

### MPV17 Mutations Affect Mitochondrial Function and the Metabolic Capacity of the Cells

**Figure 4:**
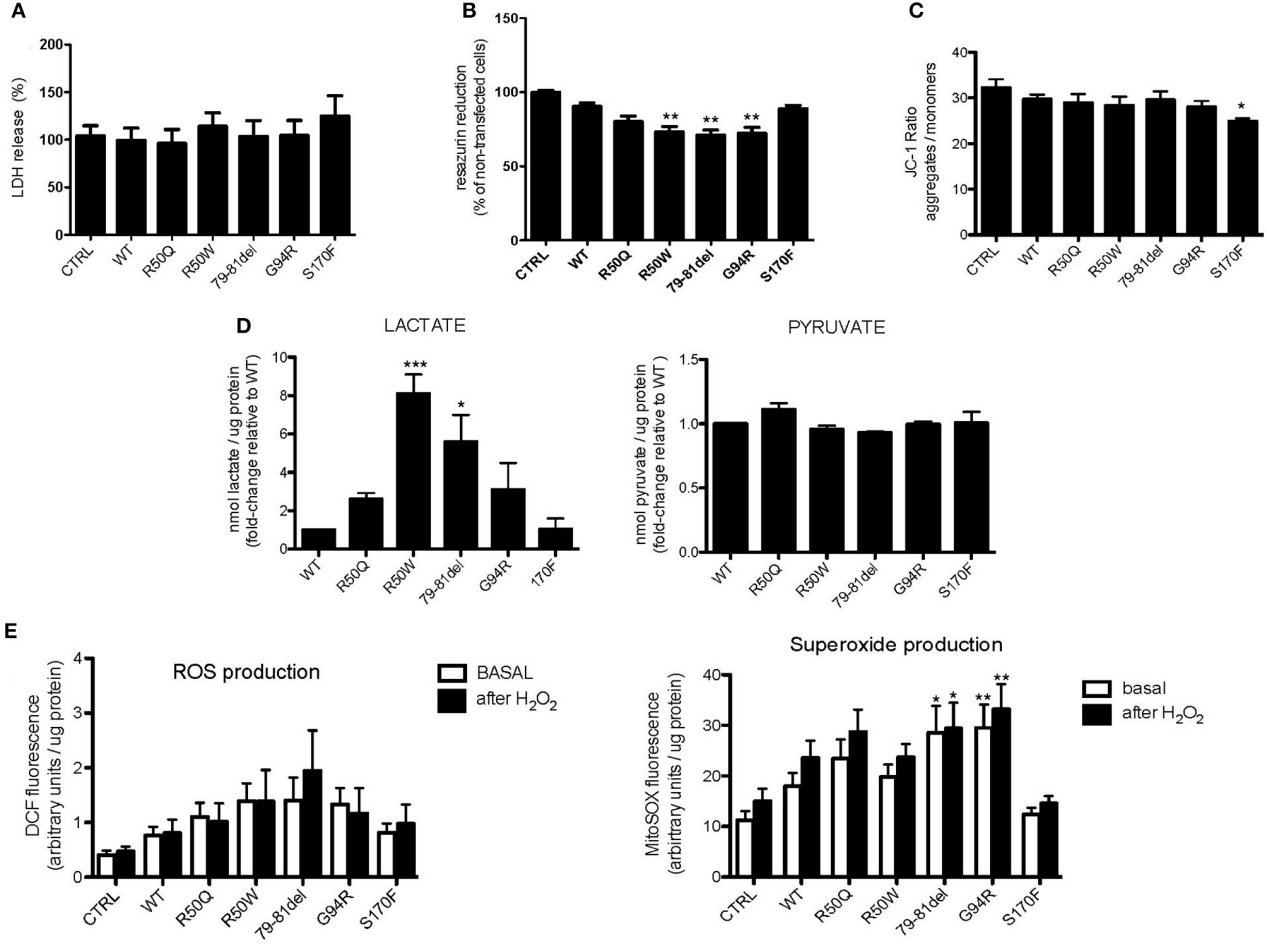
Cells expressing MPV17 mutants display mitochondrial dysfunction. MPV17^WT^ and MPV17 mutants analyzed after 48 h post-transfection. HEK293T cells were used as control (CTRL). **(A)** No difference in cell death was observed by LDH release assay in mutants when compared with MPV17^WT^. **(B)** All mutants showed decreased capacity to reduce resazurin, in particular MPV17^R50W^, MPV17^79−81del^, and MPV17^G94R^ mutants, when compared with cells expressing MPV17^WT^. **(C)** Mitochondrial membrane potential measured with JC1-probe. MPV17^S170F^ mutation caused steady-state ΔΨm loss when compared with wild-type protein. **(D)** Lactate and pyruvate measurements in 293T cells expressing the five mutants. Lactate and pyruvate levels were normalized for protein (*n* = 5) and expressed as fold-change relative to control. **(E)** Intracellular ROS and mitochondrial superoxide levels measured with DCF and MitoSOX probes, respectively, are higher in MPV17 mutants when compared with control. Stress challenge was performed by culturing cells with H_2_O_2_ 50μM for 2 h. Values represent fluorescence intensity relative to protein levels. Statistics were determined by one-way ANOVA with Bonferroni *post-hoc* test. *p < 0.05, **p < 0.01, and ***p < 0.001.

To further understand how mutations in MPV17 result in mitochondrial dysfunction, MPV17^KD^ cells were transfected with either wild-type (WT) or mutant forms of MPV17. Several parameters of mitochondrial function were assessed. We first determined whether MPV17 mutations affected cell viability/proliferation. Although we cannot completely exclude that this might be explained by differences in cell proliferation (which we could not detect by comparing cell numbers), cytotoxicity measured by LDH release did not account for the differences observed (Figure 4A). Cells expressing R50W, G79-T81del, and G94R mutations displayed decreased resazurin reduction capacity (Figure 4B). As mentioned before, this suggests either decreased cell viability and/or deficient mitochondrial oxidative capacity. Mitochondrial membrane potential (ΔΨm) is a key parameter of mitochondrial function and a useful indicator of cell viability. As suggested by microscopy experiments, the *MPV17*^G94R^ mutation appears to cause the loss of mitochondrial membrane potential. In steady-state cells, only the *MPV17*^S170F^ mutation seemed to cause significant loss of ΔΨm, as seen by decreased J-aggregates/monomers (red/green fluorescence) ratio when compared with cells expressing wild-type protein (Figure 4C). To investigate the effect of the various MPV17 forms on glycolytic activity, we measured the levels of lactate, pyruvate and the lactate/pyruvate ratio. Mutations in *MPV17* were associated with increased dependence on glycolytic ATP production (Figure 4D) and showed decreased mitochondrial competency, as evaluated by the resazurin assay. Cells expressing the *MPV17*^R50W^ mutation showed an 8-fold increase in lactate levels when compared with control cells. These differences in lactate levels were not accompanied by significant differences in pyruvate. LDH activity did not explain the differences in lactate levels between the samples (Figure 4A). Mitochondria are the major source of ROS in the cell, as a byproduct of the oxidative phosphorylation (OXPHOS) process. Thus, we assessed the possible effect of MPV17 mutations on intracellular ROS levels. We used the DCFH-DA dye in order to measure ROS levels in cells, and MitoSOX to evaluate mitochondrial superoxide (${\text{O}}_{2}^{∙}$) levels. Cells expressing most MPV17 mutants showed a tendency toward elevated levels of intracellular ROS when compared with control cells. In the case of mitochondrial superoxide, this difference was detectable both in basal conditions (with the exception of cells expressing *MPV17*^R50W^ and *MPV17*^S170F^) and in H_2_O_2_-induced stress conditions (Figure 4E).

## Discussion

MPV17 is a non-selective channel of the inner mitochondrial membrane. Its exact function as well as the mechanism leading to mtDNA instability is unknown. To gain insight into the molecular mechanisms associated with disease in patients carrying *MPV17* mutations, we evaluated the cellular consequences of MPV17 deficiency.

Lack of functional MPV17 protein in mitochondria may initiate tissue-specific cell-death and degeneration (Meyer zum Gottesberge and Felix, 2005). Our experiments indicate that MPV17^KD^ cells exhibited reduced viability while proliferation was not affected (Figure 1D) (O'Brien et al., 2000).

In the absence of MPV17, cells exhibit decreased oxygen consumption, indicating respiratory chain and OXPHOS impairment. Extracellular acidification rate (ECAR) is a surrogate measure of the production of lactic acid and, therefore, an indicator of anaerobic glycolysis. The OCR-B/ECAR ratio indicates the balance between mitochondrial respiration (OXPHOS) and glycolysis.

Frequently, defects in the former are associated with compensatory increase in the latter. As expected, OCR-B/ECAR ratio was decreased in MPV17^KD^ cells, reflecting impaired mitochondrial respiratory capacity and limited glycolytic capacity (Figures 2F,G). However, and contrary to our initial expectations, stress-induced ECAR in MPV17 deficient cells were lower than in control cells, reflecting an energetic quiescent metabolic profile. These results are consistent with those already observed in *MPV17*^−/−^ murine podocytes (Casalena et al., 2014) that showed ≈40% reduced ECAR when compared with wild-type. This is compatible with a role of MPV17 in mitochondrial homeostasis and may indicate that MPV17 is important for the activation of compensatory mechanisms in situations of impaired OXPHOS.

To date, almost 50 pathogenic mutations have been associated with *MPV17*-related MDDS. Here, we describe metabolic parameters in five cell lines expressing *MPV17* mutations, which enable the study of protein levels, localization, and effects on overall bioenergetics. Although no clear genotype-phenotype correlation currently exists, some mutations carry a better prognosis, implying that different mutations may cause distinct consequences in MPV17. Our findings demonstrate that all mutations affect the levels of the MPV17 protein (Figure 3B). Using two different techniques, we show that the *MPV17* mutations studied do not affect proper targeting of the protein to the mitochondria (Figures 3A,C). Nevertheless, MPV17^G94R^ behaved differently, as this mutant protein is found in mitochondrial enriched extract but it is not detected in the mitoplast (Figure 3D), reflecting that G94R mutation abolishes protein localization in the inner mitochondrial membrane. Although further studies are needed, one possible explanation is that the mutant protein probably is retained in the intermembrane space (IMS) or is loosely attached to the outer mitochondrial membrane (OMM).

In normally functioning mitochondria, the MPV17 channel is closed (Antonenkov et al., 2015). Nevertheless, the gating properties of the channel are modified by membrane potential, phosphorylation status and redox state. The regulation of the ΔΨm is vital to sustain mitochondrial homeostasis, as many functions like mitochondrial biogenesis, dynamics, ROS production and mitophagy depend on it. The *MPV1*7^G94R^ mutation affects ΔΨm as seen in microscopy studies using the Mitotracker Red dye. The fact that this was not obvious with the use of JC-1, another probe for measuring ΔΨm, is not clear, but might be due to different sensitivity of this probe, especially in the context of a heterogeneous population of transfected cells. The new findings regarding the effect of the G94R mutation suggests that mislocalization of MPV17 may induce MPV17 dysfunction. Moreover, together with loss of membrane potential we reasoned that this affects the protein function and is likely to play a major role in MPV17 disease in patients affected by this particular mutation.

Mutations in phosphorylation sites are responsible for several neurodegenerative disorders, including MDDS cases (Uusimaa et al., 2013). The amino acid sequence of the MPV17 protein has three predicted phosphorylation sites (Thr53, Thr80, and Ser170), and mutations in all three were found to cause MDDS. Studies employing mutants that mimic phosphorylated and unphosphorylated forms of the protein indicate that phosphorylation of MPV17 may lead to a complete closing of the channel, particularly in reducing conditions (Antonenkov et al., 2015). We demonstrate that MPV17^S170F^ causes a lower steady-state ΔΨm. However, MPV17^79−81del^, a mutation that also abolishes a phosphorylation site, does not appear the affect ΔΨm (Figure 4C). Together, this suggests that mutations on phosphorylation sites do not have a uniform pathogenic effect on the protein. Second, cells expressing the MPV17^S170F^ display lower ROS levels when compared with other mutants (Figure 4E). We hypothesize that the mutation creates a “leaky” MPV17 channel, unable to stay completely closed to preserve a normally high ΔΨm. At the same time, this may also reflect some intact compensatory mechanisms to prevent excessive ROS accumulation.

Mitochondrial DNA and proteins are particularly sensitive to ROS as they are located in close proximity to the respiratory chain. Except for MPV17^S170F^, all mutants have increased levels of mitochondrial ROS (superoxide) production. However, this increase is only significant in MPV17^79−81del^ and MPV17^G94R^ mutants. We interpret this as a common end-stage effect of different mutations, independently of the exact mechanisms by which each mutation leads to protein dysfunction. A cascade of negative effects of ROS on mtDNA stability and OXPHOS function is expected and, therefore, a common clinical phenotype of the MPV17-associated MDDS.

Although the molecular implications of our findings in simple cell systems may not be straightforward, the cellular abnormalities identified inform on possible cellular pathologies taking place. We hypothesize the effects of the mutations might be even larger in such tissues, given their high energy demand, increased number of mitochondria and dependence of OXPHOS for ATP supply.

In conclusion, we demonstrate that *MPV17* mutations can cause respiratory chain impairment by different mechanisms, including increased ROS production, decreased oxygen consumption, potential dissipation and sub-mitochondrial mislocalization. For each mutation, isolated or several simultaneous mechanisms may contribute to a cumulative deleterious effect that leads to disease. Future studies, based on our findings, will inform on the best strategy for targeting the effects of MPV17 mutations in MDDS.

## Data Availability Statement

The raw data supporting the conclusions of this article will be made available by the authors, without undue reservation.

## Author Contributions

SJ, TC-O, and TO designed the experimental work. SJ performed all the experiments, data analysis except Seahorse mitochondrial respiration studies, mtDNA copy number, and drafted the manuscript. PG performed and analyzed Seahorse mitochondrial respiration studies. MS and MG performed and interpreted mtDNA copy number. PG and RM trained SJ in performing experimental procedures and supervised the experimental work. TO and AR supervised the study and critically reviewed the manuscript. All authors reviewed, commented, and approved the manuscript.

## Conflict of Interest

The authors declare that the research was conducted in the absence of any commercial or financial relationships that could be construed as a potential conflict of interest.
