# Supplementary material for: MPV17 Mutations Are Associated With a Quiescent Energetic Metabolic Profile
Source: Front Cell Neurosci. 2021 Mar 17;15:641264. doi: 10.3389/fncel.2021.641264 (PMC8011494; doi:10.3389/fncel.2021.641264)
Supplement: Supplementary file 1 [file Table_1.DOCX]

**SUPPORTING INFORMATION**

**S1 Table**. Table of primers used for mutagenesis in human MPV17.

| MUTATION | GENOME | PRIMERS sequence (5’ → 3’) |
| --- | --- | --- |
| p.R50Q | c.149G>A | Forward: GAACACCAGAGAGGCC**A**GACTCTGACCATGGTG  Reverse: CACCATGGTCAGAGTC**T**GGCCTCTCTGGTGTTC |
| p.R50W | c.148C>T | Forward: GAACACCAGAGAGGC**T**GGACTCTGACCATGG  Reverse: CCATGGTCAGAGTCC**A**GCCTCTCTGGTGTTC |
| p.G79_T81del | c.234_242del9 | Forward: CGGTTCATCCCCAAAGTGGATGCAC  Reverse: GTGCATCCACTTTGGGGATGAACCG |
| p.G94R | c.280G>C | Forward: GTTGTTGGATCAG**C**GGGGCTTTGCC  Reverse: GGCAAAGCCCC**G**CTGATCCAACAAC |
| p.S170F | c.509C>T | Forward: GAACTCCTACCTGT**T**CTGGAAGGCACATC  Reverse: GATGTGCCTTCCAG**A**ACAGGTAGGAGTTC |
