# Supplementary material for: MPV17 Mutations Are Associated With a Quiescent Energetic Metabolic Profile
Source: Front Cell Neurosci. 2021 Mar 17;15:641264. doi: 10.3389/fncel.2021.641264 (PMC8011494; doi:10.3389/fncel.2021.641264)
Supplement: Supplementary file 2 [file Table_2.DOCX]

**SUPPORTING INFORMATION**

**S2 File.** Sequence alignment of wild-type MPV17 gene (Query) and the mutants (Sbjct) obtained by mutagenesis. In yellow is marked the nucleotide change

Mutation p.R50Q (c.149G>A) - MPV17^R50Q^

Query 1 ATGGCACTCTGGCGGGCATACCAGCGGGCCCTGGCCGCTCACCCGTGGAAAGTACAGGTC 60

||||||||||||||||||||||||||||||||||||||||||||||||||||||||||||

Sbjct 462 ATGGCACTCTGGCGGGCATACCAGCGGGCCCTGGCCGCTCACCCGTGGAAAGTACAGGTC 521

Query 61 CTGACAGCTGGGTCCCTGATGGGCCTGGGTGACATTATCTCACAGCAGCTGGTGGAGAGG 120

||||||||||||||||||||||||||||||||||||||||||||||||||||||||||||

Sbjct 522 CTGACAGCTGGGTCCCTGATGGGCCTGGGTGACATTATCTCACAGCAGCTGGTGGAGAGG 581

Query 121 CGGGGTCTGCAGGAACACCAGAGAGGCCGGACTCTGACCATGGTGTCCCTGGGCTGTGGC 180

|||||||||||||||||||||||||||| |||||||||||||||||||||||||||||||

Sbjct 582 CGGGGTCTGCAGGAACACCAGAGAGGCCAGACTCTGACCATGGTGTCCCTGGGCTGTGGC 641

Query 181 TTTGTGGGCCCTGTGGTAGGAGGCTGGTACAAGGTTTTGGATCGGTTCATCCCTGGCACC 240

||||||||||||||||||||||||||||||||||||||||||||||||||||||||||||

Sbjct 642 TTTGTGGGCCCTGTGGTAGGAGGCTGGTACAAGGTTTTGGATCGGTTCATCCCTGGCACC 701

Query 241 ACCAAAGTGGATGCACTGAAGAAGATGTTGTTGGATCAGGGGGGCTTTGCCCCGTGTTTT 300

||||||||||||||||||||||||||||||||||||||||||||||||||||||||||||

Sbjct 702 ACCAAAGTGGATGCACTGAAGAAGATGTTGTTGGATCAGGGGGGCTTTGCCCCGTGTTTT 761

Query 301 CTAGGCTGCTTTCTCCCACTGGTAGGGGCACTTAATGGACTGTCAGCCCAGGACAACTGG 360

||||||||||||||||||||||||||||||||||||||||||||||||||||||||||||

Sbjct 762 CTAGGCTGCTTTCTCCCACTGGTAGGGGCACTTAATGGACTGTCAGCCCAGGACAACTGG 821

Query 361 GCCAAACTACAGCGGGATTATCCTGATGCCCTTATCACCAACTACTATCTATGGCCTGCT 420

||||||||||||||||||||||||||||||||||||||||||||||||||||||||||||

Sbjct 822 GCCAAACTACAGCGGGATTATCCTGATGCCCTTATCACCAACTACTATCTATGGCCTGCT 881

Query 421 GTGCAGTTAGCCAACTTCTACCTGGTCCCCCTTCATTACAGGTTGGCCGTTGTCCA 476

|||||||||||||||||||||||||||||||||||||||||||||||||| |||||

Sbjct 882 GTGCAGTTAGCCAACTTCTACCTGGTCCCCCTTCATTACAGGTTGGCCGTTTCCA 936

Mutation p.R50W (c.148C>T) - MPV17^R50W^

Query 1 ATGGCACTCTGGCGGGCATACCAGCGGGCCCTGGCCGCTCACCCGTGGAAAGTACAGGTC 60

||||||||||||||||||||||||||||||||||||||||||||||||||||||||||||

Sbjct 416 ATGGCACTCTGGCGGGCATACCAGCGGGCCCTGGCCGCTCACCCGTGGAAAGTACAGGTC 475

Query 61 CTGACAGCTGGGTCCCTGATGGGCCTGGGTGACATTATCTCACAGCAGCTGGTGGAGAGG 120

||||||||||||||||||||||||||||||||||||||||||||||||||||||||||||

Sbjct 476 CTGACAGCTGGGTCCCTGATGGGCCTGGGTGACATTATCTCACAGCAGCTGGTGGAGAGG 535

Query 121 CGGGGTCTGCAGGAACACCAGAGAGGCCGGACTCTGACCATGGTGTCCCTGGGCTGTGGC 180

||||||||||||||||||||||||||| ||||||||||||||||||||||||||||||||

Sbjct 536 CGGGGTCTGCAGGAACACCAGAGAGGCTGGACTCTGACCATGGTGTCCCTGGGCTGTGGC 595

Query 181 TTTGTGGGCCCTGTGGTAGGAGGCTGGTACAAGGTTTTGGATCGGTTCATCCCTGGCACC 240

||||||||||||||||||||||||||||||||||||||||||||||||||||||||||||

Sbjct 596 TTTGTGGGCCCTGTGGTAGGAGGCTGGTACAAGGTTTTGGATCGGTTCATCCCTGGCACC 655

Query 241 ACCAAAGTGGATGCACTGAAGAAGATGTTGTTGGATCAGGGGGGCTTTGCCCCGTGTTTT 300

||||||||||||||||||||||||||||||||||||||||||||||||||||||||||||

Sbjct 656 ACCAAAGTGGATGCACTGAAGAAGATGTTGTTGGATCAGGGGGGCTTTGCCCCGTGTTTT 715

Query 301 CTAGGCTGCTTTCTCCCACTGGTAGGGGCACTTAATGGACTGTCAGCCCAGGACAACTGG 360

||||||||||||||||||||||||||||||||||||||||||||||||||||||||||||

Sbjct 716 CTAGGCTGCTTTCTCCCACTGGTAGGGGCACTTAATGGACTGTCAGCCCAGGACAACTGG 775

Query 361 GCCAAACTACAGCGGGATTATCCTGATGCCCTTATCACCAACTACTATCTATGGCCTGCT 420

||||||||||||||||||||||||||||||||||||||||||||||||||||||||||||

Sbjct 776 GCCAAACTACAGCGGGATTATCCTGATGCCCTTATCACCAACTACTATCTATGGCCTGCT 835

Query 421 GTGCAGTTAGCCAACTTCTACCTGGTCCCCCTTCATTACAGGTTGGCCG 469

|||||||||||||||||||||||||||||||||||||||||||||||||

Sbjct 836 GTGCAGTTAGCCAACTTCTACCTGGTCCCCCTTCATTACAGGTTGGCCG 884

Mutation p.G79_T81del (c.234_242del9) - MPV17^79-81del^

Query 171 GGGCTGTGGCTTTGTGGGCCCTGTGGTAGGAGGCTGGTACAAGGTTTTGGATCGGTTCAT 230

|||||||||||||||||| ||||||||||||||||||||||||||||||||||||||||

Sbjct 16 GGGCTGTGGCTTTGTGGG-CCTGTGGTAGGAGGCTGGTACAAGGTTTTGGATCGGTTCA- 73

Query 231 CCCTGGCACCACCAAAGTGGATGCACTGAAGAAGATGTTGTTGGATCAGGGGGGCTTTGC 290

| || |||||||||||||||||||||||||||||||||||||||||||||||||

Sbjct 74 ---T----CC-CCAAAGTGGATGCACTGAAGAAGATGTTGTTGGATCAGGGGGGCTTTGC 125

Query 291 CCCGTGTTTTCTAGGCTGCTTTCTCCCACTGGTAGGGGCACTTAATGGACTGTCAGCCCA 350

||||||||||||||||||||||||||||||||||||||||||||||||||||||||||||

Sbjct 126 CCCGTGTTTTCTAGGCTGCTTTCTCCCACTGGTAGGGGCACTTAATGGACTGTCAGCCCA 185

Query 351 GGACAACTGGGCCAAACTACAGCGGGATTATCCTGATGCCCTTATCACCAACTACTATCT 410

||||||||||||||||||||||||||||||||||||||||||||||||||||||||||||

Sbjct 186 GGACAACTGGGCCAAACTACAGCGGGATTATCCTGATGCCCTTATCACCAACTACTATCT 245

Query 411 ATGGCCTGCTGTGCAGTTAGCCAACTTCTACCTGGTCCCCCTTCATTACAGGTTGGCCGT 470

||||||||||||||||||||||||||||||||||||||||||||||||||||||||||||

Sbjct 246 ATGGCCTGCTGTGCAGTTAGCCAACTTCTACCTGGTCCCCCTTCATTACAGGTTGGCCGT 305

Query 471 TGTCCAATGTGTTGCTGTTATCTGGAACTCCTACCTGTCCTGGAAGGCACATCGGCTCTA 530

||||||||||||||||||||||||||||||||||||||||||||||||||||||||||||

Sbjct 306 TGTCCAATGTGTTGCTGTTATCTGGAACTCCTACCTGTCCTGGAAGGCACATCGGCTCTA 365

Mutation p.G94R (c.280G>C) - MPV17^G94R^

Query 171 GGGCTGTGGCTTTGTGGGCCCTGTGGTAGGAGGCTGGTACAAGGTTTTGGATCGGTTCAT 230

||||||||||||||||||||||||||||||||||||||||||||||||||||||||||||

Sbjct 11 GGGCTGTGGCTTTGTGGGCCCTGTGGTAGGAGGCTGGTACAAGGTTTTGGATCGGTTCAT 69

Query 231 CCCTGGCACCACCAAAGTGGATGCACTGAAGAAGATGTTGTTGGATCAGGGGGGCTTTGC 290

||||||||||||||||||||||||||||||||||||||||||||||||| ||||||||||

Sbjct 70 CCCTGGCACCACCAAAGTGGATGCACTGAAGAAGATGTTGTTGGATCAGCGGGGCTTTGC 129

Query 291 CCCGTGTTTTCTAGGCTGCTTTCTCCCACTGGTAGGGGCACTTAATGGACTGTCAGCCCA 350

||||||||||||||||||||||||||||||||||||||||||||||||||||||||||||

Sbjct 130 CCCGTGTTTTCTAGGCTGCTTTCTCCCACTGGTAGGGGCACTTAATGGACTGTCAGCCCA 189

Query 351 GGACAACTGGGCCAAACTACAGCGGGATTATCCTGATGCCCTTATCACCAACTACTATCT 410

||||||||||||||||||||||||||||||||||||||||||||||||||||||||||||

Sbjct 190 GGACAACTGGGCCAAACTACAGCGGGATTATCCTGATGCCCTTATCACCAACTACTATCT 249

Query 411 ATGGCCTGCTGTGCAGTTAGCCAACTTCTACCTGGTCCCCCTTCATTACAGGTTGGCCGT 470

||||||||||||||||||||||||||||||||||||||||||||||||||||||||||||

Sbjct 250 ATGGCCTGCTGTGCAGTTAGCCAACTTCTACCTGGTCCCCCTTCATTACAGGTTGGCCGT 309

Query 471 TGTCCAATGTGTTGCTGTTATCTGGAACTCCTACCTGTCCTGGAAGGCACATCGGCTCTA 530

||||||||||||||||||||||||||||||||||||||||||||||||||||||||||||

Sbjct 310 TGTCCAATGTGTTGCTGTTATCTGGAACTCCTACCTGTCCTGGAAGGCACATCGGCTCTA 369

Mutation p.S170F (c.509C>T) - MPV17^S170F^

Query 1 ATGGCACTCTGGCGGGCATACCAGCGGGCCCTGGCCGCTCACCCGTGGAAAGTACAGGTC 60

||||||||||||||||||||||||||||||||||||||||||||||||||||||||||||

Sbjct 518 ATGGCACTCTGGCGGGCATACCAGCGGGCCCTGGCCGCTCACCCGTGGAAAGTACAGGTC 577

Query 61 CTGACAGCTGGGTCCCTGATGGGCCTGGGTGACATTATCTCACAGCAGCTGGTGGAGAGG 120

||||||||||||||||||||||||||||||||||||||||||||||||||||||||||||

Sbjct 578 CTGACAGCTGGGTCCCTGATGGGCCTGGGTGACATTATCTCACAGCAGCTGGTGGAGAGG 637

Query 121 CGGGGTCTGCAGGAACACCAGAGAGGCCGGACTCTGACCATGGTGTCCCTGGGCTGTGGC 180

||||||||||||||||||||||||||||||||||||||||||||||||||||||||||||

Sbjct 638 CGGGGTCTGCAGGAACACCAGAGAGGCCGGACTCTGACCATGGTGTCCCTGGGCTGTGGC 697

Query 181 TTTGTGGGCCCTGTGGTAGGAGGCTGGTACAAGGTTTTGGATCGGTTCATCCCTGGCACC 240

||||||||||||||||||||||||||||||||||||||||||||||||||||||||||||

Sbjct 698 TTTGTGGGCCCTGTGGTAGGAGGCTGGTACAAGGTTTTGGATCGGTTCATCCCTGGCACC 757

Query 241 ACCAAAGTGGATGCACTGAAGAAGATGTTGTTGGATCAGGGGGGCTTTGCCCCGTGTTTT 300

||||||||||||||||||||||||||||||||||||||||||||||||||||||||||||

Sbjct 758 ACCAAAGTGGATGCACTGAAGAAGATGTTGTTGGATCAGGGGGGCTTTGCCCCGTGTTTT 817

Query 301 CTAGGCTGCTTTCTCCCACTGGTAGGGGCACTTAATGGACTGTCAGCCCAGGACAACTGG 360

||||||||||||||||||||||||||||||||||||||||||||||||||||||||||||

Sbjct 818 CTAGGCTGCTTTCTCCCACTGGTAGGGGCACTTAATGGACTGTCAGCCCAGGACAACTGG 877

Query 361 GCCAAACTACAGCGGGATTATCCTGATGCCCTTATCACCAACTACTATCTATGGCCTGCT 420

||||||||||||||||||||||||||||||||||||||||||||||||||||||||||||

Sbjct 878 GCCAAACTACAGCGGGATTATCCTGATGCCCTTATCACCAACTACTATCTATGGCCTGCT 937

Query 421 GTGCAGTTAGCCAACTTCTACCTGGTCCCCCTTCATTACAGGTTGGCCGTTGTCCAATGT 480

||||||||||||||||||||||||||||||||||||||||||||||||||||||||||||

Sbjct 938 GTGCAGTTAGCCAACTTCTACCTGGTCCCCCTTCATTACAGGTTGGCCGTTGTCCAATGT 997

Query 481 GTTGCTGTTATCTGGAACTCCTACCTGTCCTGGAAGGCACATCGGCTCT 529

||||||||||||||||||||||||||||| |||||||||||||| ||||

Sbjct 998 GTTGCTGTTATCTGGAACTCCTACCTGTCTTGGAAGGCACATCG-CTCT 1044
