# Supplementary material for: MPV17 Mutations Are Associated With a Quiescent Energetic Metabolic Profile
Source: Front Cell Neurosci. 2021 Mar 17;15:641264. doi: 10.3389/fncel.2021.641264 (PMC8011494; doi:10.3389/fncel.2021.641264)
Supplement: Supplementary file 3 [file Presentation_1.PPTX]

## Slide 1
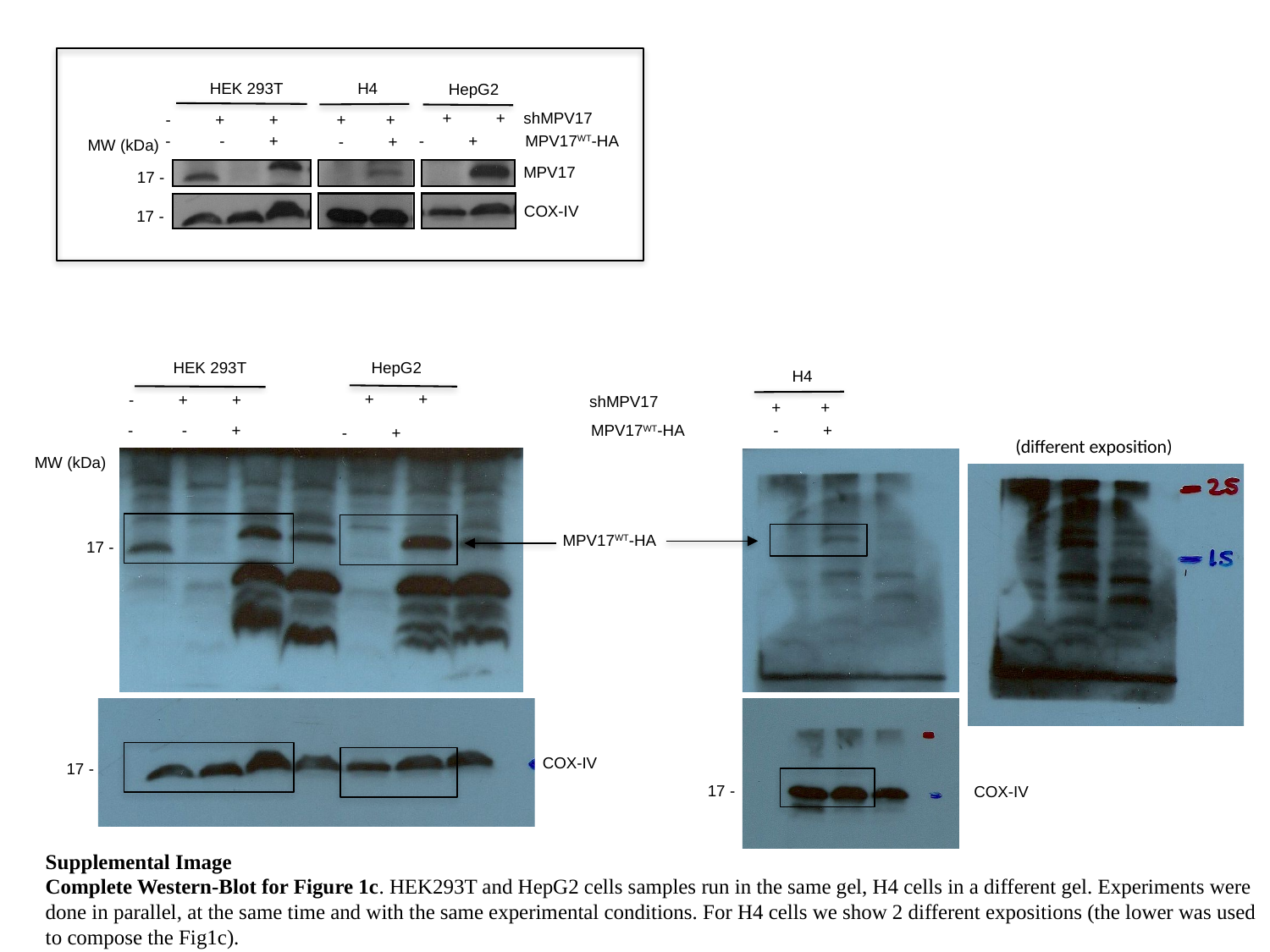

HEK 293T
H4
HepG2
shMPV17
 + +
 - + +
 + +
 - +
MPV17WT-HA
 - - +
 - +
MW (kDa)
MPV17
17 -
COX-IV
17 -
HEK 293T
HepG2
H4
 + +
 - + +
shMPV17
 + +
 - +
 - - +
MPV17WT-HA
 - +
(different exposition)
MW (kDa)
MPV17WT-HA
17 -
COX-IV
17 -
17 -
COX-IV
Supplemental Image
Complete Western-Blot for Figure 1c. HEK293T and HepG2 cells samples run in the same gel, H4 cells in a different gel. Experiments were done in parallel, at the same time and with the same experimental conditions. For H4 cells we show 2 different expositions (the lower was used to compose the Fig1c).
